# Sex, Ecology and the Brain: Evolutionary Correlates of Brain Structure Volumes in Tanganyikan Cichlids

**DOI:** 10.1371/journal.pone.0014355

**Published:** 2010-12-17

**Authors:** Alejandro Gonzalez-Voyer, Niclas Kolm

**Affiliations:** 1 Department of Integrative Ecology, Estación Biológica de Doñana – Consejo Superior de Investigación Científica (CSIC), Sevilla, Spain; 2 Animal Ecology, Department of Evolutionary Biology, Evolutionary Biology Centre, Uppsala University, Uppsala, Sweden; University of Lethbridge, Canada

## Abstract

Analyses of the macroevolutionary correlates of brain structure volumes allow pinpointing of selective pressures influencing specific structures. Here we use a multiple regression framework, including phylogenetic information, to analyze brain structure evolution in 43 Tanganyikan cichlid species. We analyzed the effect of ecological and sexually selected traits for species averages, the effect of ecological traits for each sex separately and the influence of sexual selection on structure dimorphism. Our results indicate that both ecological and sexually selected traits have influenced brain structure evolution. The patterns observed in males and females generally followed those observed at the species level. Interestingly, our results suggest that strong sexual selection is associated with reduced structure volumes, since all correlations between sexually selected traits and structure volumes were negative and the only statistically significant association between sexual selection and structure dimorphism was also negative. Finally, we previously found that monoparental female care was associated with increased brain size. However, here cerebellum and hypothalamus volumes, after controlling for brain size, associated negatively with female-only care. Thus, in accord with the mosaic model of brain evolution, brain structure volumes may not respond proportionately to changes in brain size. Indeed selection favoring larger brains can simultaneously lead to a reduction in relative structure volumes.

## Introduction

There is compelling evidence across very diverse species that both ecological and social factors can play an important role in shaping brain evolution [1], [2], [3], [4]. Increasing demands on cognitive ability predominantly alter the size of neural structures rather than their connectivity [5], [6]. Hence, relative brain size and gross brain structure constitute measurable reflections of the way a species has adapted to a given environmental context or selection regime [7], [8], [9]. Phylogenetic comparative analyses have proven particularly useful to identify macroevolutionary correlates of brain evolution across very diverse species. In mammals, social living appears to have played a key role in shaping brain evolution and larger brains have been linked with invasion success and longer lifespan [4], [10], [11], [12], [13], [14]. In birds, larger brains have been associated with tool use, survival, invasion success and developmental mode [15], [16], [17]. Finally, although less studied than mammals or birds, available evidence in fishes shows an association between ecological niche, parental care (biparental or monoparental care) and brain size [18], [19].

Although changes in whole brain size necessarily reflect selection acting on one or multiple structures within the brain, particular brain structures may not respond proportionally to changes in whole brain size [3]. And there could also be trade-offs between brain areas that cannot be measured in whole brain size [20]. Indeed, studies in mammals, birds and fish suggest that brain structures evolve, to a certain extent, in a mosaic fashion and increases or decreases in size of particular brain structures can occur independently of changes in other structures [8], [21], [22], [23]. Hence, while analyses of whole brain size should reveal the action of selection leading to measurable changes in brain size, analyses of the correlates of structure volume may allow for pinpointing more specific selective pressures influencing particular structures, which might not reflect on changes in whole brain size [20]. For example, diurnal mammals possess a larger visual cortex than nocturnal ones, and neocortex size in primates is positively correlated to social group size [4], [24]. Wing area, a proxy for habitat complexity, correlates positively with a sub-cortical auditory centre (inferior colliculi) in echolocating bats, and with the hippocampus in all bats [25]. In birds, initial analyses suggested brain size was positively associated with innovation rate, but closer examination showed that the best predictor of this behavior was the relative size of an association area in the forebrain [the mesopallium ventrale; 26]. Also, comparative analyses have shown that the higher vocal centre is significantly associated with song complexity [27], [28] while male brain size did not correlate significantly with song complexity [29]. Studies of brain structure evolution have also allowed identification of evolutionary convergence such as the association between large relative hippocampal size and i) food storing in mammals and birds, ii) brood parasitism in birds, and iii) large home-range size in mammals and birds (reviewed in [30]). Finally, analyses of brain structure in fishes suggest that it is influenced by diet, habitat complexity and life-history [2], [9], [19], [31], [32], [33]. And results from one of these studies suggest that, as with mammals and birds, social factors can influence structure volume [19].

The cognitive demands associated with locating and competing for mates, as well as mate selection, could potentially lead to sexual dimorphism in brain structure [34]. Spatial abilities might confer an advantage in mate location, enhanced motor control could be advantageous during physical contests or displays, and if cognitive ability can be accurately assessed then it could become a sexually selected trait [34], [35]. There is increasing evidence supporting the hypothesis of sexual selection acting as an evolutionary force shaping brain size and structure, although there are also contradictory results. For instance, males in polygynous meadow voles (*Microtus pennsylvanicus*) have a significantly larger hippocampus than conspecific females, whereas no sexual dimorphism is evident in closely related monogamous pine voles (*M. pinetorum*) [36]. In carnivores, females providing sole parental care have larger brains than those of biparental or communal species [37]. A similar pattern was recently found in Tanganyikan cichlids [18], where results indicated that sexual selection influences parental care patterns [38]. In brown trout (*Salmo trutta*), where males compete intensely for females, males possess a larger telencephalon [33]. Passerine species with larger inter-sexual differences in song complexity also present larger dimorphism in brain size between the sexes [29]. Furthermore, in bird species with a higher degree of extra-pair paternity females had larger brains than conspecific males, whereas in species with lower rates of extra-pair paternity brain size dimorphism was male biased [39]. However, a study with waterfowl found no evidence of sexual dimorphism in brain size associated with sperm competition or pair bond duration [40]. And a study with mammals found no relationship between brain size and testis mass [41]. To date, relatively few studies have analyzed brain structure evolution in both sexes and a recent study highlights the pitfalls of analyzing sexually selected characters independently, without including previously identified ecological correlates [42].

Here we analyze brain structure evolution in 43 species of Tanganyikan cichlid fish. Tanganyikan cichlids are an excellent model to study brain structure evolution as they are the most diverse phenotypically, morphologically and behaviorally of the African cichlids and recent morphological analyses have demonstrated the adaptive nature of their radiation [43], [44]. Because individual structures sometimes overlap in function and each structure can have more than one function [3], it is difficult to make precise predictions about how ecology and sexual selection might correlate with brain structure volumes. However, based on theory and existing information from previous comparative analyses [9], [18], [19], [32] we can make the following predictions. Given the roles of the telencephalon and cerebellum in processing information from the surrounding environment, particularly with regards to spatial cognition and spatial learning [45], [46], [47], we predict that the volumes of these two structures should be positively correlated with habitat complexity but negatively correlated to depth [19]. For olfactory bulbs and optic tecta, we predict, again based on the assumption that deeper habitats contain less visual information, that olfactory bulbs should be positively associated to depth while optic tecta should show the opposite pattern and be negatively correlated to depth [9]. In line with theory and previous empirical evidence [18], [34], we predict positive associations, at least in males but possibly also for species means, between the intensity of sexual selection and brain structures related to visual and olfactory processing (optic tecta and olfactory bulbs), spatial orientation (telencephalon, cerebellum) and coordination of movements (cerebellum), all potentially important components of both male-male competition and female mate-choice [34], [48]. Based on previous results showing a sex-specific effect of parental care type on brain size [18], we predict a positive association between telencephalon volume and monoparental care in females, assuming that the effect on brain size is due to increased cognitive demands resulting from monoparental care of offspring. Finally, in accordance with the social brain hypothesis [11], we again build on previous results from analyses of total brain size in cichlid fish [18], which found that species feeding on algae had larger brain size. We have previously suggested this is due to that the niche occupied by algae-eaters is also the one where most social interactions occur, both within and between species [18]. Hence, we predict a similar link between telencephalon size (and possibly also for olfactory bulbs and optic tecta) and diet. Note that we have not made a-priori predictions for all structures due to the above-mentioned difficulties.

Our sample included sexually mature male and female individuals allowing us to analyze both species-specific as well as sex-specific effects. We used a multiple regression approach, controlling for phylogenetic effects [20], [49], to analyze the influence of ecology, behavior, and sexually selected traits. In accord with this, results indicate that both ecological and sexually selected traits are significantly associated brain structure volumes and that sex-specific patterns generally followed those observed at the species level. Surprisingly, all correlations between brain structure volume and sexually selected traits were negative suggesting the possibility of a trade-off between sexually selected traits and cognitive ability.

## Results

### Species-specific correlates

**Figure 1:**
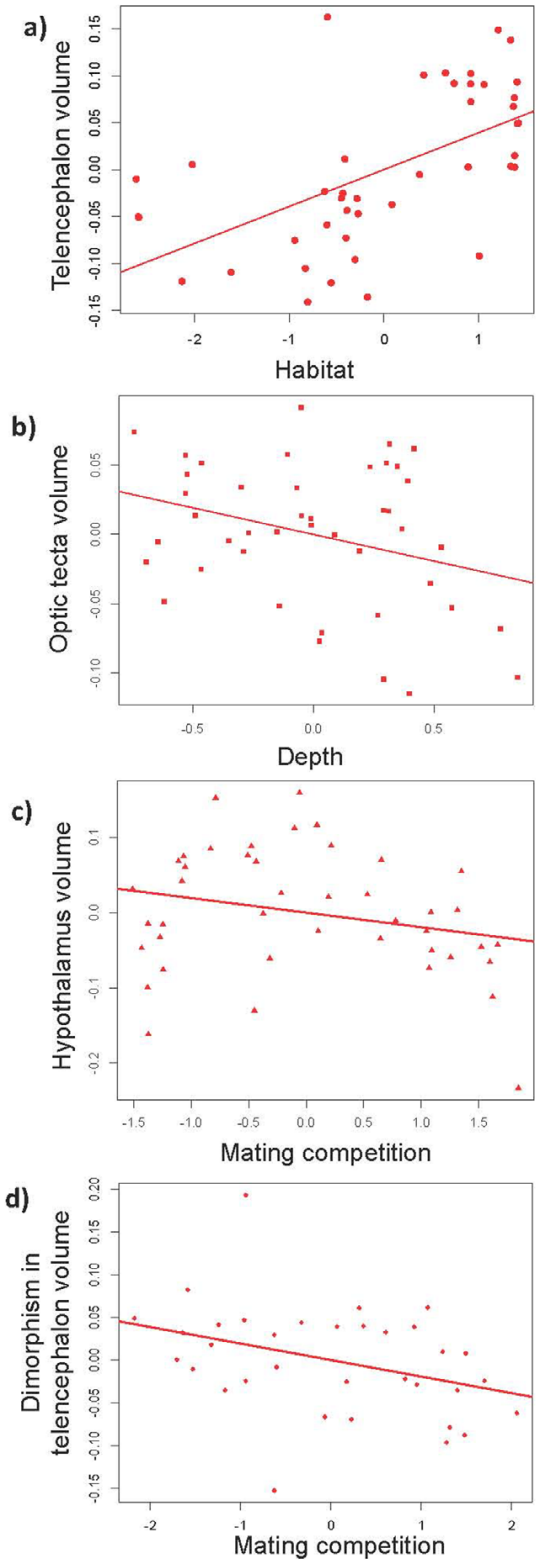
Ecological and sexually selected correlates of brain structure volumes. Partial regression graphs of the relationship between relative brain structure volumes (when controlling for brain size; see Methods for details) and ecological characters or sexual traits. Partial regression relationships were obtained from a linear regression model without controlling for phylogeny and are shown for illustration purposes only. a) Relative telencephalon volume as a function of habitat; b) relative optic tecta volume as a function of depth; c) relative hypothalamus volume as a function of mating competition and d) relative sexual dimorphism in telencephalon volume as a function of mating competition.

The high values of the evolutionary parameter (λ) in the pgls models (with the exception of optic tecta) indicate that the covariance between brain structures and their correlates evolves following Brownian motion (Table 1). Olfactory bulb volume correlated negatively with habitat complexity, indicating that species living in less complex habitats (e. g. benthic or sand) have larger olfactory bulbs than species from complex (e. g. rock) habitats (Table 1). On the contrary, telencephalon volume was positively correlated with habitat complexity, indicating that species inhabiting rocky habitats have a larger telencephalon than species from benthic or sandy habitats (Table 1; Fig. 1, a). Optic tecta volume was significantly negatively correlated with depth (Table 1; Fig. 1, b). Interestingly, the covariance between depth and optic tecta volume does not follow a Brownian motion model since the lambda value was equal to 0. Cerebellum volume was significantly correlated with sexual selection, depth and habitat (Table 1). Sexual selection was negatively associated with cerebellum volume (sexually selected traits loaded negatively on the PC, see Methods), while the relationship with depth and habitat was positive. When we tried to tease apart the effect of sexual selection, neither mating competition nor sexual dimorphism was significantly correlated with cerebellum volume on their own (p = 0.10 and p = 0.18, respectively), hence it appears the effect is mediated by a combination of the sexually selected traits. In accord with this, care type was negatively correlated with cerebellum volume (β = −0.088±0.021, p = 0.0002). Dorsal medulla volume was negatively correlated with mating competition (Table 1). Finally, hypothalamus volume was also negatively correlated with mating competition (Table 1; Fig. 1, c). In accord with this, the hypothalamus was also negatively correlated with care type (β = −0.066±0.028, p = 0.02).

**Table 1:** Correlates of brain structure volume for the species-specific measures.

**Olfactory bulbs**	λ = 1	
Brain	1.05±0.09	p<0.0001
Habitat	−0.053±0.012	p = 0.0001
**Telencephalon**	λ = 0.78	
Brain	1.087±0.046	p<0.0001
Habitat	0.038±0.009	p = 0.0002
**Optic tecta**	λ = 0	
Brain	1.059±0.038	p<0.0001
Depth	−0.038±0.018	p = 0.04
**Cerebellum**	λ = 1	
Brain	1.196±0.043	p<0.0001
Sexual selection	0.032±0.009	p = 0.0007
Depth	0.061±0.027	p = 0.028
Habitat	0.031±0.010	p = 0.0025
**Dorsal medulla**	λ = 1	
Brain	1.118±0.101	p<0.0001
Mating competition	−0.0455±0.0190	p = 0.02
**Hypothalamus**	λ = 1	
Brain	1.078±0.056	p<0.0001
Mating competition	−0.022±0.011	p = 0.046

For each model we present the value of the evolutionary parameter of the gls model, the partial regression slopes, standard error and p-values. Only correlates retained in the minimum adequate model are shown.

### Sex-specific correlates

The covariance between brain structures and their correlates presented distinct evolutionary patterns between males and females, as evidenced by the differences in the values of the evolutionary parameters of the pgls models (Table 2). As would be expected, the results of the sex-specific analyses generally confirmed those of the species level analyses. In both males and females habitat was negatively correlated with olfactory bulb volume but positively correlated with telencephalon volume (Table 2). Furthermore, as in the species level analyses, we found a negative correlation between optic tecta volume and depth in both sexes, again with a null value of lambda suggesting non-Brownian covariance between these traits (Table 2). However, there were differences between the sexes in the correlates of structure volume, notably for the cerebellum and dorsal medulla (Table 2). Cerebellum volume was negatively correlated with depth in females only. On the other hand, male cerebellum volume correlated negatively with care type (β = −0.057±0.029, p = 0.05). Female cerebellum volume was not significantly correlated with care type (β = −0.021±0.026, p = 0.44). The dorsal medulla was negatively correlated with habitat complexity in females only (Table 2).

**Table 2:** Sex-specific, ecological brain structure correlates.

Males			Females		
**Olfactory bulbs**	λ = 0.68			λ = 0.99	
Brain	0.950±0.168	p<0.0001	Brain	0.894±0.012	p<0.0001
Habitat	−0.07±0.032	p = 0.047	Habitat	−0.068±0.02	p = 0.002
**Telencephalon**	λ = 0.80			λ = 0.86	
Brain	1.04±0.05	p<0.0001	Brain	1.14±0.06	p<0.0001
Habitat	0.04±0.01	p = 0.0002	Habitat	0.03±0.011	p = 0.025
**Optic tecta**	λ = 0			λ = 0	
Brain	1.07±0.04	p<0.0001	Brain	1.05±0.04	p<0.0001
Depth	−0.05±0.02	p = 0.03	Depth	−0.07±0.02	p = 0.001
**Cerebellum**	λ = 0			λ = 0.3	
Brain	1.12±0.051	p<0.0001	Brain	1.17±0.05	p<0.0001
			Depth	−0.06±0.028	p = 0.026
**Dorsal Medulla**	λ = 1			λ = 1	
Brain	0.87±0.15	p<0.0001	Brain	0.95±0.11	p<0.0001
			Habitat	−0.04±0.01	p<0.0001
**Hypothalamus**	λ = 0.74			λ = 1	
Brain	0.95±0.057	p<0.0001	Brain	0.99±0.07	p<0.0001

For each model we present the value of the evolutionary parameter of the gls model, the partial regression coefficients and their standard errors, as well as the associated p-value. Only correlates retained in the minimum adequate model are shown.

### Brain structure dimorphism

The evolution of brain structure dimorphism showed notable departure from Brownian motion as shown by the low values of the evolutionary parameter (Table 3). A significant effect of sexual selection on brain structure dimorphism was found only for the telencephalon, although the optic tecta presented a marginally non-significant effect (Table 3). Our results indicate that as the intensity of mating competition increases, sexual dimorphism in telencephalon volume decreases (Fig. 1, d). The optic tecta presented a non-signficant (p = 0.069) trend in the opposite direction. Finally, care type was also negatively correlated with sexual dimorphism in telencephalon volume (β = −0.060±0.024, t = −2.50, p = 0.02).

**Table 3:** Sexually selected characters associated with sexual dimorphism in brain structure volume.

**Olfactory bulbs**	λ = 0	
Brain	1.00±0.32	p = 0.004
**Telencephalon**	λ = 0	
Brain	1.25±0.15	p<0.0001
Mating competition	−0.02±0.01	p = 0.04
**Optic tecta**	λ = 0.09	
Brain	0.86±0.07	p<0.0001
Mating competition	0.008±0.004	p = 0.07
**Cerebellum**	λ = 0.01	
Brain	0.84±0.14	p<0.0001
**Dorsal medulla**	λ = 0.87	
Brain	0.99±0.31	p = 0.004
**Hypothalamus**	λ = 0	
Brain	0.91±0.11	p<0.0001

For each model we present the value of the evolutionary parameter of the gls model, the partial regression coefficients and their standard errors, as well as the associated p-value.

## Discussion

### Species-specific correlates

A combination of ecological variables as well as sexually selected traits correlated significantly with structure volumes. Our results thus suggest that different selective forces influence the evolution of the distinct structures within the brain, in line with the mosaic model of brain evolution [8], [21], [22], [23]. Furthermore, our results suggest that there could be trade-offs between structure volumes resulting from contrasting selective forces derived from a single trait. An example of such contrasting selection is apparent in the association between habitat and three distinct structures, the olfactory bulbs, telencephalon and cerebellum. In line with our predictions, habitat complexity correlated positively with telencephalon and cerebellum volume, while olfactory bulb volume was negatively correlated with habitat complexity. An earlier study, focusing on seven species from a monophyletic group of Tanganyikan cichlids, also found a positive association between habitat complexity and telencephalon and cerebellum volume [19]. Hence, our results suggest complex habitats select for species with a larger telencephalon and cerebellum, while species inhabiting less complex habitats (e. g. benthic habitats) rely more on olfactory cues. Fish, like amniotes are able to use cognitive mapping strategies to navigate to a goal, and experiments have shown that the Teleost telencephalon has specific functions in spatial learning and memory [50], [51]. The cerebellum is not only essential for modulating the planning and execution of motor activity as experiments have also shown that this structure is important in various learning and memory processes associated with spatial orientation [50]. Hence, it is possible that habitat complexity favors species with a larger telencephalon and cerebellum through demands on spatial cognition and spatial memory. Alternatively, the association between telencephalon, cerebellum and habitat complexity could also be mediated through social factors, rather than purely ecological effects. A previous study has shown that both species richness and density of individuals increases with habitat complexity, and both variables correlated positively with telencephalon and cerebellum volume [19]. Brain size was also previously suggested to covary with social complexity [18].

Again in line with our predictions, depth was negatively correlated with optic tecta volume, which is in agreement with results from a previous study with African cichlids that also found a negative association between depth and optic tecta volume [9]. Because phylogenetic information was not available at the time, Huber *et al* [9] were unable to include it in their analyses. Interestingly, the null value of lambda suggests that the covariance between these traits does not proceed according to a Brownian motion model, which suggests that there is a minor influence of shared ancestry on the relationship between these traits, or that evolution has occurred rapidly, eroding the phylogenetic signal [52]. On the other hand, our initial prediction of a negative correlation between olfactory bulb volume and depth was not supported.

The cerebellum was the only brain structure to present significant correlations with both ecological and sexually selected traits. Cerebellum volume increased with depth and with habitat complexity, as predicted, and decreased with increasingly intense sexual selection, contrary to our prediction. These results highlight the interplay between sexual selection and environmental characteristics of a species' niche. Sexual selection is not independent of the environment. On the contrary, a species' ecological niche can influence the mating system and secondary sexual signals which may develop [53]. The Lamprologini tribe of Tanganyikan cichlids provides a nice example of this since species have been categorized as permanently or temporarily haremic, bigamous, or monogamous and the mating system and degree of sexual size dimorphism appear to be related to the number of suitable spawning sites within a male's territory [54]. However, we did not find any signal for sexual selection leading to sexual size dimorphism in cerebellum volume, suggesting that the effect is similar in both sexes.

Both the dorsal medulla and the hypothalamus presented significantly negative associations with mating competition, indicating that species with more intense precopulatory and postcopulatory mating competition have smaller dorsal medulla and hypothalamus. Pollen *et al*. [19] found that polygamous species had a larger hypothalamus than monogamous species, which is contrary to our results. The difference could result from the fact that these authors included only 7 Tanganyikan species, which presented only two independent evolutionary transitions in mating system (see [19], p 33, Fig. 7).

We had predicted a positive association between telencephalon volume and diet, based on previous results indicating that species feeding on sessile prey (aufwuchs and algae) had larger brains than species feeding on more motile prey [18], however the prediction was not supported by our data. We speculate that the larger brain size associated with species feeding on sessile prey results from a combination of the positive correlation between telencephalon and cerebellum volumes and habitat complexity as well as the negative correlation between optic tecta volume and depth. This suggestion is supported by the fact that sessile prey is generally located in the complex rocky habitat, which in turn tends to be in shallow waters [55].

### Sex-specific correlates

The results of the sex-specific analyses generally supported the findings of the species-specific analyses. However, there were also cases where the selective forces acting on male and female structures differed. For the olfactory bulbs, telencephalon and optic tecta, the correlates were the same both at the species level and between the two sexes. Habitat complexity correlated significantly with olfactory bulbs and telencephalon volumes, although the direction of the relationship was opposite for the two structures. The olfactory bulbs were previously found to be the most variable structure, in comparison with all the others, with respect to changes in total brain size [23]; and our results suggest that such variability may be the result of adaptation to different ecological niches. The same appears to be the case for the telencephalon, previously found to be the most variable structure among cichlid species from the three African Lakes [32], although that study did not incorporate phylogenetic information. The optic tecta showed a significant negative correlation with depth in both sexes. On the other hand, cerebellum volume correlated negatively with depth but only in females, and the dorsal medulla correlated negatively with habitat in females only. Cerebellum volume was negatively correlated with care type, but only significantly so in males (i. e. in species with female only care, males had smaller cerebellum volumes). It is difficult to disentangle whether the effect was caused by sexual selection or care type since these two traits are highly correlated [38]. Finally, the hypothalamus was only correlated significantly with brain size and this was the case in both sexes.

### Sexual selection and brain structure volume

Sexual dimorphism in structure volume was only apparent for the telencephalon, where mating competition was significantly negatively correlated with telencephalon dimorphism. The optic tecta showed a marginally non-significant trend in the opposite direction. This result is in line with available evidence that suggests the telencephalon is larger in monogamous than polygamous Tanganyikan cichlids [19]. Sex differences in telencephalon volume have also been found in brown trout, with males presenting a larger telencephalon than females [33].

Contrary to our initial predictions based on previous studies having found a positive association between strength of sexual selection and structure or brain volume (e. g. [29]), our results suggest that brain structure volumes decrease with increasing strength of sexual selection. Regardless of whether the effect was mediated through mating competition, or a combination of mating competition and sexual dimorphism, the correlation between sexually selected traits and structure volume was always negative. A previous study has suggested that strong sexual selection could lead to a reduction in brain size. Pitnick et al. [56] found that bat species with promiscuous females have relatively smaller brains than do species were females exhibit mate fidelity. The authors suggested that the relationship resulted from the negative evolutionary relationship between investment in two expensive tissues, brains and testes. However, a later study found that the relationship between testis size and brain size disappeared when morphological adaptation to foraging strategy is included in the analyses [42]. Our results suggest that in Tanganyikan cichlids strong sexual selection can result in reduced structure volume. Mating system in Tanganyikan cichlids is strongly correlated with sexual selection [38]. An earlier study found evidence suggesting that monogamous Tanganyikan species had a larger telencephalon than polygamous species [19]. However this comparison involved only 3 monogamous and 4 polygamous species and there were only two independent evolutionary changes in mating system. In contrast, our results indicate that increased mating competition leads to a decrease in sexual dimorphism in telencephalon volume. The results from both studies can be reconciled under a scenario where the increase in telencephalon volume in monogamous species (presenting reduced mating competition) is the result of accentuated sexual dimorphism in telencephalon volume. In Tanganyikan cichlids it is mostly males that invest in mate competition [38], [57]. It is possible that intense mating competition bears costs to males, which potentially limit investment in expensive brain tissue [58], [59]. Alternatively, increased mating competition among males could select for choosier females, which under such circumstances only gain fitness benefits from their choice of mate through good genes or sexy-sons effects [48]. Finally, it is important to note that intensity of sexual selection and parental care are correlated in Tanganyikan cichlids [38]. Indeed, the cerebellum, hypothalamus and sexual dimorphism in telencephalon volume all correlated significantly with mating system as well as with care type. At this point it is thus not possible to determine whether the effect is due to sexual selection, care type of a combination of the two. Further analyses, which are beyond the scope of this study, might allow us to disentangle the effect of these two intercorrelated variables.

### Whole brain size vs structure volumes

There was one notable difference between the results obtained when analyzing whole brain size versus when we analyzed structure volumes. The difference lies in the association with care type: while in the whole brain size analyses we found that species in which females cared for offspring alone had larger brains [18], here we found that such species had a smaller cerebellum and a smaller hypothalamus (controlling for total brain size). Hence, while female only care of offspring has apparently selected for larger brains, results suggest that such species have a smaller cerebellum and hypothalamus for their brain size. This result lends further support to previous analyses suggesting that Tanganyikan cichlid brains evolve following a mosaic model [23], as the relative volumes of the cerebellum and hypothalamus do not increase with increasing brain size, rather the contrary. However, the sex-specific analyses present a slightly different picture. The cerebellum was the only structure to correlate significantly with care type when the analyses were repeated separately for each sex. Cerebellum volume was negatively correlated with care type in males only. This suggests that the negative correlation observed at the species level could in part be due to a decrease in cerebellum volume in males, which would tend to lower the species average. We would need to increase the sample size for the sex-specific sample to be able to obtain a clearer picture of the influence of parental care on structure volumes.

Finally, the contrasting pattern observed between whole brain size, brain structure volume and care type suggest that caution must be exerted when attempting to relate the results of analyses of whole brain size to what may be occurring to brain structures [20]. Larger brains might not necessarily result in increased relative volumes in all structures and, as shown here, may even involve in some cases a reduction in relative structure volume.

## Methods

Ethics statement: The study was approved by the Uppsala Animal Research Ethical Board; permit number (C264/6).

### Data

We obtained volumetric measures of brain structures for 43 Tanganyikan cichlid species (see [23] for details of sampled species and sample sizes). Our sample included most Tanganyikan species for which detailed phylogenetic information is available, and provides a representative sample of natural variation in the lake, including 7 out of the 12 tribes into which Tanganyikan cichlids have been grouped [44]. Data is provided as online supplementary Material S1.

Brains were collected from wild caught, sexually mature individuals. Fish were first deeply anesthetized with benzocaine and then the head was severed and preserved in 4% paraformaldehyde in a phosphate buffer for tissue fixation and preservation. Whole brain weight (±0.001 g) was obtained from dissected brains following fixation (see [23] for further details). Intraspecific sample sizes = 3–7 individuals, except for two species for which we only had one sample.

**Figure 2:**
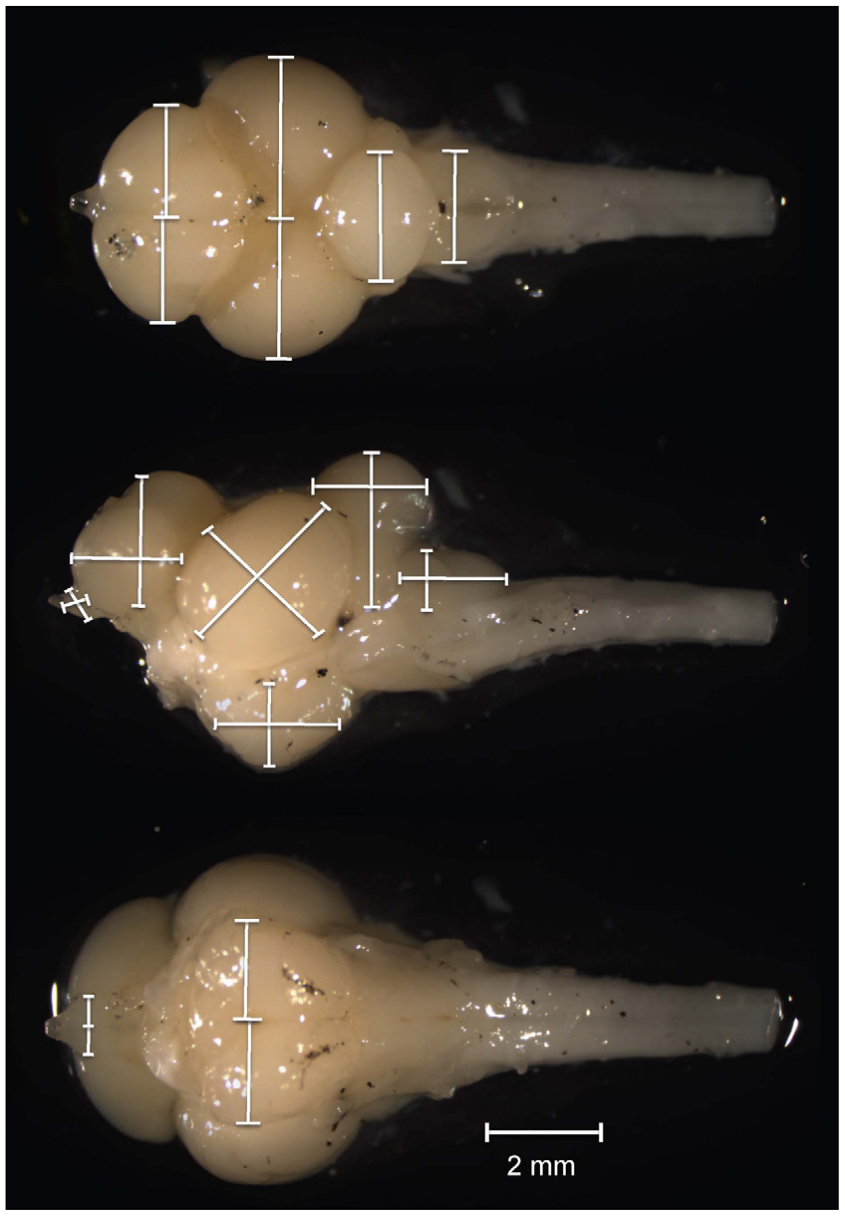
Dorsal, ventral and lateral views of a Tanganyikan cichlid brain. Shown are the measures (length, width and height) that were taken for each of the 6 brain structures (olfactory bulbs, telencephalon, optic tecta, cerebellum, dorsal medulla and hypothalamus). See Methods for further details.

All dissections, digital images and measurements were performed by the same person (AG-V). All were done blindly since specimens were identified by number and not species name. Digital images of the dorsal, ventral, left and right sides of the brain were taken through a dissection microscope (Leica MZFLIII), using a digital camera (Leica DFC 490 and Firecam v. 3.1 software). For each image the brain was carefully placed on a Petri dish with 0.9% agar, which was solid but would yield to brains and allow for them to be placed in such a manner to ensure that the view of the brain being photographed was horizontal and both sides were symmetrical. For paired structures, both were measured and the volume was the sum of the two structures. We followed the procedure of Pollen et al. [19] to measure length, width and height of six key-structures: olfactory bulbs, telencephalon, optic tecta, cerebellum, hypothalamus and dorsal medulla (see Fig. 2 for measure illustrations). The volume of each structure was quantified according to the ellipsoid model: V = (L×W×H) π/6 which provides consistent estimates of the volume of brain structures in Taganyikan cichlids [9], [19], [32] even when compared to volumes obtained from slices [19]. To estimate repeatability the volume of all structures was measured twice on one randomly picked specimen from each of the 43 species. In all cases the correlation coefficient between repeated measures for all structures was high, r>0.98. To verify that intraspecific variability was similar among structures, we compared the species-specific standard errors across the 6 structures. There was no significant difference in standard error between structures (F = 1.91, p = 0.09, df = 5, 257; none of the post-hoc analyses were significant: range of p-values = 0.22–1.00), suggesting that there is no systematic bias. The summed volume of the 6 measured structures provided a reliable estimate of total brain size as the summed volume correlated strongly with brain weight (r = 0.96). All data was log_10_ transformed and because some of the measures were smaller than 1, we multiplied all data by 1000 prior to log transformation [60].

Diet and habitat were coded as continuous variables representing variation in prey motility and habitat complexity. Qualitative descriptions of both variables were transformed into quantitative continuous variables reflecting a continuum of variation. Diet reflected variation in prey motility, with sessile prey such as aufwuchs and fixed algae at one extreme and fishes at the other (for further details see [18]). Habitat reflected variation in complexity: benthic and benthopelagic habitats were the least complex and rocky habitats the most complex (for further details see [18]). It has been previously shown that such categorical ranking of habitats captures significant variation in quantitative measures of complexity [19]. Most species do not strictly inhabit a single habitat or feed on one prey type; therefore we used descriptive information on habitat preferences and prey to calculate an average for each species giving more weight to preferred habitats/prey based on detailed descriptions from primary publications (see [18]). Form of care was coded as a dichotomous variable representing mouthbrooding or substrate guarding, while care type was coded as a dichotomous variable representing biparental or female-only care (as in [18]). Data on depth was collected from Konings [55], as well as from FishBase, and by contacting researchers studying particular species when no published data was available. Prevalence of sperm competition was ranked (1–4) following Fitzpatrick et al [61], based on information about mating system and fertilization location; which have been previously found to correlate significantly with different sperm characteristics [61]. Mating system was coded as in Seehausen et al [62] to reflect intensity of precopulatory sexual selection. Ranks varied from 1 to 4, although they are taken to reflect a continuum of variation, with monogamous species at one extreme and promiscuous species, e.g. lekking, at the other extreme. Sexual dichromatism and sexual shape dimorphism were ranked independently by four Tanganyikan cichlid experts. For each species the experts were asked whether the sexes presented differences in coloration or shape (independently of size dimorphism); both variables were coded as dichotomous reflecting presence or absence of sexual differences. Disagreement between the experts was limited to the ranks for sexual shape dimorphism of 4 species. In these rare instances, we used the rank of the expert who had most experience observing the species in their natural habitat (data is available as online supplementary Material S1). Mating system and sperm competition are correlated (Tsuboi et al. unpublished data), thus to avoid multicolinearity problems, they were combined into a single variable (henceforth mating competition) reflecting the combination of pre and postcopulatory competition using phylogenetic principal components analysis (PCCA; [63]). Sexual dichromatism and shape dimorphism were also combined into a single variable (henceforth sexual dimorphism) using PPCA. Finally, we combined all sexually selected traits into a single variable (henceforth sexual selection) using PPCA. Note that all sexually selected traits loaded negatively on the first component in this PPCA (loadings: mating system = −0.79, sperm competition = −0.87, sexual dichromatism = −0.75 and shape dimorphism = −0.65). Below, when referring to the relationship between sexual selection and structure volume we will present it based on these negative loadings on the PC, such that the relationship with the measures of sexual selection is the correct one. In the multiple regression models (see below) we first included the variable sexual selection; if there was a marginally significant signal of sexual selection we tried to disentangle the effects by including separately – in a stepwise fashion – mating competition or sexual dimorphism.

### Phylogeny

We reconstructed a molecular phylogeny for the 49 species included in the analyses using mitochondrial sequences downloaded from Genbank under Bayesian inference [64] in MrBayes v3.1 [65]. We used two coding sequences, cytochrome b and NADH2, and one non-coding gene, the control region, which were concatenated to create a matrix of 1819 base pairs. Coding sequences were partitioned by codon and the analyses were run using a GTR+I+γ model of substitution selected using jModel test [66]. We ran 7 million iterations of the Markov chain sampling every 1 000^th^ iteration with burnin at 1 750 000 iterations. Convergence was confirmed using AWTY [67]. The molecular phylogeny was cropped to include the 43 species for the species-specific analyses and 33 species for the sex-specific analyses. Branch lengths reflecting number of expected substitutions were included in all analyses.

### Phylogenetic comparative analyses

Correlates of brain structure evolution were identified by means of phylogenetic generalized least squares (pgls) multiple regression models [68]. Analyses were undertaken using the package *ape* [69] in R [70]. In all cases the maximum likelihood value of the evolutionary parameter (λ), which resulted in the variance co-variance matrix approximating a Brownian motion model of evolution [52], was estimated simultaneously with the multiple regression model [71]. Models were constructed by including all ecological variables, form of care and sexual selection, as independent variables, and as a co-variate [72] we included brain weight, the dependent variable in each model was the volume of the brain structure. Care type was analyzed separately from sexual selection as they are highly correlated [38]. First, we created models including species averages for each brain structure as the dependent variable. We then repeated the analyses separately for each sex. We also calculated sexual dimorphism in structure volume and brain weight using the formula: Log(male structure volume/female structure volume) [73] and analyses were repeated using sexual dimorphism in structure volume as the dependent variable.

For illustration purposes we present 4 graphs showing the relationship between specific relative structure volumes and ecological characters or sexual traits. Note that the graphs represent the relationships without controlling for phylogeny and that they were created based on a least squares linear model. Plots describe the partial regression relationship between structure volume, after controlling for allometric effects with brain size, and either an ecological character or sexual traits, for which the association with brain size, if any, is controlled (see Fig. 1). Plots were created in R using package car.

## Supporting Information

Material S1 Brain structure volumes, ecological characters and sexually selected traits.
(0.25 MB DOC)
