# Supplementary material for: Sex, Ecology and the Brain: Evolutionary Correlates of Brain Structure Volumes in Tanganyikan Cichlids
Source: PLoS One. 2010 Dec 17;5(12):e14355. doi: 10.1371/journal.pone.0014355 (PMC3003682; doi:10.1371/journal.pone.0014355)
Supplement: Material S1 — Brain structure volumes, ecological characters and sexually selected traits. (0.25 MB DOC) [file pone.0014355.s001.doc]

S1. Brain structure volumes, ecological characters and sexually selected traits.

| Species | Olfactory bulbs | Telencephalon | Optic tecta | Cerebellum | Dorsal medula | Hypothalamus | Brain weight |
| --- | --- | --- | --- | --- | --- | --- | --- |
| Altolamprologus compressiceps | 1.3568 | 3.9101 | 4.2856 | 3.7997 | 2.8127 | 3.7483 | 1.8375 |
| Aulonocranus dewindti | 1.1750 | 3.7649 | 4.2912 | 3.7828 | 3.1682 | 3.6678 | 1.9012 |
| Benthochromis tricoti | 1.3605 | 3.9264 | 4.5129 | 4.0402 | 3.2545 | 3.7168 | 2.0642 |
| Cyphotilapia frontosa | 1.6960 | 4.4360 | 4.6735 | 4.3358 | 3.4376 | 4.1640 | 2.3054 |
| Cyathopharynx furcifer | 1.4535 | 4.0482 | 4.5857 | 4.1002 | 3.4791 | 3.9181 | 2.1388 |
| Ctenochromis horei | 1.8734 | 4.1544 | 4.6087 | 4.0935 | 3.2637 | 3.9990 | 2.1971 |
| Cyprichromis leptosoma | 1.1400 | 3.8439 | 4.3276 | 3.8407 | 2.8367 | 3.6200 | 1.8617 |
| Callochromis melanostigma | 1.2493 | 3.5317 | 4.0225 | 3.5435 | 3.2523 | 3.4802 | 1.6041 |
| Cyprichromis microlepidotus | 1.2728 | 4.0234 | 4.4944 | 4.0997 | 3.1278 | 3.7907 | 2.0675 |
| Callochromis pleurospilus | 1.4393 | 3.6681 | 4.1737 | 3.7133 | 3.3169 | 3.6132 | 1.7961 |
| Eretmodus cyanostictus | 1.2975 | 3.6059 | 4.0382 | 3.6082 | 2.7241 | 3.4451 | 1.5767 |
| Enantiopus melanogenys | 1.4267 | 3.9029 | 4.4409 | 4.0415 | 3.5925 | 3.8679 | 2.0286 |
| Greenwoodochromis christyi | 1.4964 | 3.9714 | 4.3598 | 4.0044 | 2.9356 | 3.7280 | 1.9888 |
| Gnathochromis permaxillaris | 1.4457 | 3.8858 | 4.2574 | 3.8849 | 3.2985 | 3.6793 | 1.9220 |
| Haplotaxodon microlepis | 1.4862 | 3.8049 | 4.4616 | 3.9644 | 3.1711 | 3.5906 | 1.9645 |
| Julidochromis marlieri | 1.2705 | 3.7806 | 4.0796 | 3.6628 | 2.5959 | 3.6324 | 1.6704 |
| Julidochromis ornatus | 1.3182 | 3.7342 | 3.9561 | 3.6062 | 2.4539 | 3.5051 | 1.6790 |
| Julidochromis regani | 1.3567 | 3.8465 | 4.1154 | 3.6905 | 2.6311 | 3.6731 | 1.7202 |
| Julidochromis transcriptus | 1.1724 | 3.5533 | 3.8759 | 3.4567 | 2.3487 | 3.3559 | 1.5481 |
| Limnochromis auritus | 1.1921 | 3.6266 | 4.0036 | 3.5248 | 2.8294 | 3.4142 | 1.7131 |
| Lamprologus callipterus | 1.2465 | 3.5457 | 4.1252 | 3.4804 | 2.7002 | 3.4277 | 1.6756 |
| Limnotilapia dardennii | 1.3299 | 3.6517 | 4.2163 | 3.6458 | 2.8642 | 3.5463 | 1.7585 |
| Lepidiolamprologus nkambae | 1.2490 | 3.8997 | 4.3633 | 3.7802 | 2.8825 | 3.6862 | 1.9114 |
| Neolamprologus brevis | 0.6718 | 3.1879 | 3.6685 | 2.9102 | 1.9765 | 2.9471 | 1.3619 |
| Neolamprologus brichardi | 1.1729 | 3.6124 | 3.9419 | 3.4830 | 2.5571 | 3.4534 | 1.5558 |
| Neolamprologus tetracanthus | 1.4974 | 3.9636 | 4.4675 | 3.8207 | 2.9082 | 3.7332 | 2.0086 |
| Neolamprologus tretocephalus | 1.2959 | 3.8153 | 4.2537 | 3.8104 | 3.0216 | 3.6787 | 1.8426 |
| Ophthalmotilapia boops | 0.8528 | 3.8918 | 4.3324 | 3.8448 | 3.1636 | 3.5941 | 1.9156 |
| Ophthalmotilapia nasuta | 1.0080 | 3.9269 | 4.3751 | 3.8611 | 3.2428 | 3.6995 | 1.9566 |
| Ophthalmotilapia ventralis | 1.1805 | 4.0897 | 4.4858 | 4.0148 | 3.2866 | 3.8299 | 2.0427 |
| Paracyprichromis brieni | 0.7817 | 3.5900 | 3.9589 | 3.4872 | 2.5898 | 3.4089 | 1.6717 |
| Petrochromis famula | 1.7726 | 4.2903 | 4.6666 | 4.2836 | 3.3952 | 4.1416 | 2.2945 |
| Petrochromis orthognathus | 1.7127 | 4.3773 | 4.6625 | 4.3094 | 3.3603 | 4.0397 | 2.2933 |
| Simochromis babaulti | 1.5127 | 3.8267 | 4.2954 | 3.7512 | 2.7358 | 3.6091 | 1.8915 |
| Spathodus erythrodon | 1.2636 | 3.6417 | 4.0134 | 3.5566 | 2.6182 | 3.4645 | 1.5824 |
| Spathodus marlieri | 1.2174 | 3.6527 | 4.0084 | 3.5353 | 2.6536 | 3.4047 | 1.6433 |
| Tropheus brichardi | 1.5973 | 4.1996 | 4.5446 | 4.2089 | 3.0759 | 4.0169 | 2.1251 |
| Tanganicodus irsacae | 1.0878 | 3.6508 | 4.0472 | 3.5276 | 2.7540 | 3.4762 | 1.6014 |
| Tropheus moorii | 1.2675 | 3.9932 | 4.3405 | 3.9221 | 2.8582 | 3.7158 | 1.9416 |
| Triglachromis otostigma | 1.2678 | 3.5670 | 4.0387 | 3.5530 | 2.8020 | 3.3846 | 1.6568 |
| Xenotilapia flavipinnis | 0.8929 | 3.3904 | 3.9197 | 3.3732 | 3.1875 | 3.3819 | 1.4848 |
| Xenotilapia ochrogenys | 1.4351 | 3.6308 | 4.1245 | 3.6482 | 3.6339 | 3.6675 | 1.8036 |
| Xenotilapia spilopterus | 1.0131 | 3.6746 | 4.1514 | 3.6055 | 3.4390 | 3.5963 | 1.7672 |

**Table S1.** Brain structure volumes and whole brain weight for species averages (See methods for details on measures and transformations).

**Table S2.** Female brain structure volumes and whole brain weight (See methods for details on measures and transformations).

| Species | Olfactory bulbs | Telencephalon | Optic tecta | Cerebellum | Dorsal medula | Hypothalamus | Brain weight |
| --- | --- | --- | --- | --- | --- | --- | --- |
| Altolamprologus compressiceps | 2.3247 | 3.9071 | 4.2693 | 3.7864 | 2.8048 | 3.7393 | 1.8132 |
| Aulonocranus dewindti | 2.2828 | 3.8310 | 4.2943 | 3.8294 | 3.2068 | 3.6696 | 1.9069 |
| Cyphotilapia frontosa | 2.6649 | 4.3948 | 4.6624 | 4.2901 | 3.4332 | 4.1334 | 2.2879 |
| Cyathopharynx furcifer | 2.4549 | 4.0816 | 4.5805 | 4.0547 | 3.4598 | 3.9297 | 2.1417 |
| Ctenochromis horei | 2.6491 | 3.9653 | 4.4142 | 3.9058 | 3.1246 | 3.8562 | 2.0453 |
| Cyprichromis leptosoma | 2.1524 | 3.7590 | 4.2910 | 3.7905 | 2.8883 | 3.5417 | 1.8141 |
| Callochromis melanostigma | 2.2206 | 3.5291 | 4.0135 | 3.5264 | 3.2745 | 3.4525 | 1.5919 |
| Eretmodus cyanostictus | 2.2922 | 3.4881 | 3.9847 | 3.5459 | 2.5857 | 3.3314 | 1.5051 |
| Enantiopus melanogenys | 2.5397 | 3.8470 | 4.3510 | 3.8917 | 3.5578 | 3.7692 | 1.9542 |
| Greenwoodochromis christyi | 2.5646 | 4.0388 | 4.3764 | 4.0162 | 3.1786 | 3.7424 | 2.0289 |
| Gnathochromis permaxillaris | 2.5241 | 4.0156 | 4.3374 | 3.9582 | 3.3216 | 3.7774 | 2.0062 |
| Haplotaxodon microlepis | 2.5151 | 3.7628 | 4.4676 | 3.9133 | 3.0132 | 3.6060 | 1.9563 |
| Julidochromis marlieri | 2.3023 | 3.7824 | 4.0873 | 3.6758 | 2.5715 | 3.6368 | 1.6778 |
| Julidochromis ornatus | 2.3107 | 3.7044 | 3.9087 | 3.5379 | 2.4711 | 3.5272 | 1.6765 |
| Julidochromis regani | 2.4057 | 3.8652 | 4.1332 | 3.7164 | 2.6655 | 3.6900 | 1.7349 |
| Julidochromis transcriptus | 2.1734 | 3.5485 | 3.8804 | 3.4258 | 2.3302 | 3.3480 | 1.5335 |
| Limnochromis auritus | 2.1122 | 3.6290 | 4.0087 | 3.4913 | 2.8460 | 3.3912 | 1.7130 |
| Limnotilapia dardennii | 2.4009 | 3.6441 | 4.2112 | 3.6477 | 2.9135 | 3.5941 | 1.7882 |
| Lepidiolamprologus nkambae | 2.1764 | 3.7833 | 4.3727 | 3.8206 | 2.9029 | 3.6582 | 1.9243 |
| Neolamprologus brichardi | 2.1773 | 3.6035 | 3.9428 | 3.4770 | 2.5525 | 3.4507 | 1.5537 |
| Neolamprologus tretocephalus | 2.3492 | 3.8243 | 4.2434 | 3.8035 | 3.0547 | 3.6643 | 1.8392 |
| Ophthalmotilapia boops | 1.9795 | 3.8753 | 4.2990 | 3.8494 | 3.1434 | 3.5363 | 1.8779 |
| Ophthalmotilapia nasuta | 1.9429 | 3.8741 | 4.3246 | 3.7936 | 3.1908 | 3.6667 | 1.9138 |
| Ophthalmotilapia ventralis | 2.0819 | 4.0742 | 4.4662 | 3.9701 | 3.2349 | 3.7731 | 2.0165 |
| Paracyprichromis brieni | 1.7883 | 3.5662 | 3.9519 | 3.4680 | 2.6375 | 3.3800 | 1.6568 |
| Petrochromis orthognathus | 2.6645 | 4.3949 | 4.6517 | 4.2887 | 3.3168 | 4.0298 | 2.2879 |
| Simochromis babaulti | 2.4794 | 3.8055 | 4.2672 | 3.7127 | 2.6936 | 3.5442 | 1.8630 |
| Spathodus erythrodon | 2.2432 | 3.6015 | 4.0099 | 3.5637 | 2.3950 | 3.4601 | 1.5682 |
| Spathodus marlieri | 2.2061 | 3.6220 | 3.9867 | 3.5007 | 2.6258 | 3.3902 | 1.6231 |
| Tropheus brichardi | 2.5745 | 4.2067 | 4.5396 | 4.1887 | 3.0909 | 4.0214 | 2.1266 |
| Tanganicodus irsacae | 2.0933 | 3.6042 | 4.0654 | 3.5098 | 2.7375 | 3.4450 | 1.6008 |
| Tropheus moorii | 2.2909 | 3.9294 | 4.3020 | 3.8773 | 2.8035 | 3.6939 | 1.8886 |
| Triglachromis otostigma | 2.2499 | 3.5691 | 4.0546 | 3.5460 | 2.7875 | 3.3710 | 1.6663 |
| Xenotilapia flavipinnis | 2.0012 | 3.3974 | 3.9330 | 3.3950 | 3.1862 | 3.4120 | 1.5185 |

**Table S3.** Male brain structure volumes and whole brain weight (See methods for details on measures and transformations).

| Species | Olfactory bulbs | Telencephalon | Optic tecta | Cerebellum | Dorsal medula | Hypothalamus | Brain weight |
| --- | --- | --- | --- | --- | --- | --- | --- |
| Altolamprologus compressiceps | 2.5171 | 3.9254 | 4.3670 | 3.8659 | 2.8521 | 3.7933 | 1.9590 |
| Aulonocranus dewindti | 2.1031 | 3.7318 | 4.2896 | 3.7595 | 3.1489 | 3.6668 | 1.8983 |
| Cyphotilapia frontosa | 2.7270 | 4.4979 | 4.6902 | 4.4042 | 3.4443 | 4.2099 | 2.3317 |
| Cyathopharynx furcifer | 2.4521 | 4.0148 | 4.5910 | 4.1457 | 3.4984 | 3.9065 | 2.1359 |
| Ctenochromis horei | 2.9855 | 4.2489 | 4.7060 | 4.1873 | 3.3333 | 4.0704 | 2.2730 |
| Cyprichromis leptosoma | 2.1276 | 3.9288 | 4.3643 | 3.8909 | 2.7851 | 3.6984 | 1.9093 |
| Callochromis melanostigma | 2.2780 | 3.5344 | 4.0315 | 3.5606 | 3.2302 | 3.5080 | 1.6163 |
| Eretmodus cyanostictus | 2.2993 | 3.6452 | 4.0560 | 3.6289 | 2.7703 | 3.4830 | 1.6006 |
| Enantiopus melanogenys | 2.3890 | 3.9215 | 4.4709 | 4.0914 | 3.6041 | 3.9008 | 2.0534 |
| Greenwoodochromis christyi | 2.3600 | 3.8365 | 4.3266 | 3.9810 | 2.4495 | 3.6990 | 1.9085 |
| Gnathochromis permaxillaris | 2.3672 | 3.7560 | 4.1773 | 3.8116 | 3.2754 | 3.5812 | 1.8377 |
| Haplotaxodon microlepis | 2.4717 | 3.8259 | 4.4586 | 3.9899 | 3.2501 | 3.5829 | 1.9686 |
| Julidochromis marlieri | 2.1117 | 3.7716 | 4.0412 | 3.5978 | 2.7178 | 3.6104 | 1.6335 |
| Julidochromis ornatus | 2.3220 | 3.7491 | 3.9798 | 3.6403 | 2.4453 | 3.4941 | 1.6802 |
| Julidochromis regani | 2.2588 | 3.8092 | 4.0796 | 3.6387 | 2.5623 | 3.6393 | 1.6908 |
| Julidochromis transcriptus | 2.1704 | 3.5631 | 3.8668 | 3.5183 | 2.3858 | 3.3717 | 1.5774 |
| Limnochromis auritus | 2.3518 | 3.6219 | 3.9934 | 3.5919 | 2.7961 | 3.4602 | 1.7131 |
| Limnotilapia dardennii | 2.2943 | 3.6554 | 4.2189 | 3.6449 | 2.8396 | 3.5225 | 1.7437 |
| Lepidiolamprologus nkambae | 2.2635 | 3.9230 | 4.3614 | 3.7722 | 2.8784 | 3.6918 | 1.9088 |
| Neolamprologus brichardi | 2.1468 | 3.6661 | 3.9359 | 3.5191 | 2.5843 | 3.4698 | 1.5682 |
| Neolamprologus tretocephalus | 2.2559 | 3.8085 | 4.2615 | 3.8157 | 2.9968 | 3.6895 | 1.8450 |
| Ophthalmotilapia boops | 1.5993 | 3.9248 | 4.3990 | 3.8358 | 3.2042 | 3.7097 | 1.9912 |
| Ophthalmotilapia nasuta | 2.0732 | 3.9797 | 4.4257 | 3.9285 | 3.2948 | 3.7323 | 1.9995 |
| Ophthalmotilapia ventralis | 2.2791 | 4.1053 | 4.5053 | 4.0596 | 3.3383 | 3.8866 | 2.0689 |
| Paracyprichromis brieni | 1.7751 | 3.6137 | 3.9659 | 3.5063 | 2.5420 | 3.4377 | 1.6865 |
| Petrochromis orthognathus | 2.8572 | 4.3245 | 4.6947 | 4.3717 | 3.4911 | 4.0694 | 2.3096 |
| Simochromis babaulti | 2.5628 | 3.8585 | 4.3376 | 3.8089 | 2.7990 | 3.7066 | 1.9342 |
| Spathodus erythrodon | 2.2705 | 3.6551 | 4.0146 | 3.5543 | 2.6926 | 3.4660 | 1.5871 |
| Spathodus marlieri | 2.2401 | 3.7140 | 4.0520 | 3.6045 | 2.7093 | 3.4335 | 1.6838 |
| Tropheus brichardi | 2.6659 | 4.1785 | 4.5597 | 4.2696 | 3.0307 | 4.0034 | 2.1206 |
| Tanganicodus irsacae | 2.0824 | 3.6973 | 4.0290 | 3.5454 | 2.7706 | 3.5075 | 1.6021 |
| Tropheus moorii | 2.2441 | 4.0570 | 4.3790 | 3.9669 | 2.9128 | 3.7378 | 1.9947 |
| Triglachromis otostigma | 2.3035 | 3.5629 | 4.0071 | 3.5670 | 2.8311 | 3.4119 | 1.6378 |
| Xenotilapia flavipinnis | 1.8568 | 3.3880 | 3.9152 | 3.3659 | 3.1879 | 3.3719 | 1.4736 |

**Table S4.** Sexually selected traits and ecological characters used in the comparative analyses.

| Species | Sexual dichromatism | Shape dimorphism | Mating system | Sperm competition | Depth | Diet | Habitat | Form of care | Parental care |
| --- | --- | --- | --- | --- | --- | --- | --- | --- | --- |
| Altolamprologus compressiceps | 1 | 1 | 2 | 3 | 1.2430 | 4.50 | 4.26 | 0 | 1 |
| Aulonocranus dewindti | 2 | 1 | 4 | 3 | 0.7404 | 3.50 | 2.00 | 1 | 1 |
| Benthochromis tricoti | 2 | 2 | 2 | 3 | 1.4771 | 3.50 | 1.50 | 1 | 1 |
| Cyphotilapia frontosa | 1 | 2 | 3 | 3 | 1.7404 | 4.33 | 5.00 | 1 | 1 |
| Cyathopharynx furcifer | 2 | 2 | 4 | 4 | 1.0414 | 3.70 | 2.70 | 1 | 1 |
| Ctenochromis horei | 2 | 1 | 3 | 3 | 0.7782 | 4.00 | 3.20 | 1 | 1 |
| Cyprichromis leptosoma | 2 | 1 | 2 | 4 | 1.3324 | 4.50 | 3.20 | 1 | 1 |
| Callochromis melanostigma | 2 | 1 | 2 | 3 | 0.3979 | 3.00 | 3.30 | 1 | 1 |
| Cyprichromis microlepidotus | 2 | 1 | 4 | 4 | 1.3979 | 4.50 | 3.20 | 1 | 1 |
| Callochromis pleurospilus | 2 | 1 | 2 | 3 | 0.3010 | 3.00 | 3.30 | 1 | 1 |
| Eretmodus cyanostictus | 1 | 1 | 1 | 1 | 0.1761 | 2.50 | 4.50 | 1 | 0 |
| Enantiopus melanogenys | 2 | 2 | 4 | 4 | 1.4393 | 4.00 | 2.80 | 1 | 1 |
| Greenwoodochromis christyi | 1 | 1 | 1 | 2 | 1.5441 | 5.50 | 1.60 | 1 | 0 |
| Gnathochromis permaxillaris | 1 | 1 | 1 | 2 | 1.8129 | 5.00 | 1.00 | 1 | 0 |
| Haplotaxodon microlepis | 1 | 1 | 1 | 2 | 1.0000 | 5.00 | 3.45 | 1 | 0 |
| Julidochromis marlieri | 1 | 1 | 1 | 3 | 1.2430 | 3.33 | 4.80 | 0 | 0 |
| Julidochromis ornatus | 1 | 1 | 1 | 4 | 1.3522 | 5.00 | 5.00 | 0 | 0 |
| Julidochromis regani | 1 | 1 | 1 | 2 | 0.6990 | 4.50 | 3.00 | 0 | 0 |
| Julidochromis transcriptus | 1 | 1 | 1 | 2 | 1.1761 | 5.00 | 5.00 | 0 | 0 |
| Limnochromis auritus | 1 | 1 | 1 | 1 | 1.8129 | 5.00 | 1.00 | 1 | 0 |
| Lamprologus callipterus | 2 | 1 | 2 | 4 | 0.8451 | 4.67 | 4.60 | 0 | 1 |
| Limnotilapia dardennii | 2 | 1 | 4 | 2 | 1.3979 | 1.60 | 3.20 | 1 | 1 |
| Lepidiolamprologus nkambae | 1 | 1 | 1 | 2 | 1.3802 | 5.50 | 5.00 | 0 | 0 |
| Neolamprologus brevis | 2 | 1 | 2 | 4 | 0.8808 | 4.00 | 3.00 | 0 | 1 |
| Neolamprologus brichardi | 1 | 1 | 1 | 3 | 1.0000 | 3.33 | 4.00 | 0 | 0 |
| Neolamprologus tetracanthus | 1 | 1 | 2 | 3 | 0.6021 | 4.33 | 3.00 | 0 | 1 |
| Neolamprologus tretocephalus | 1 | 1 | 1 | 2 | 1.0000 | 3.33 | 4.50 | 0 | 0 |
| Ophthalmotilapia boops | 2 | 2 | 4 | 4 | 0.5441 | 2.50 | 5.00 | 1 | 1 |
| Ophthalmotilapia nasuta | 2 | 2 | 4 | 4 | 1.0000 | 4.00 | 4.00 | 1 | 1 |
| Ophthalmotilapia ventralis | 2 | 2 | 4 | 4 | 0.7782 | 2.50 | 5.00 | 1 | 1 |
| Paracyprichromis brieni | 2 | 2 | 4 | 3 | 1.2430 | 4.00 | 3.20 | 1 | 1 |
| Petrochromis famula | 1 | 1 | 4 | 2 | 0.5441 | 1.00 | 5.00 | 1 | 1 |
| Petrochromis orthognathus | 1 | 1 | 4 | 3 | 1.0212 | 1.00 | 4.40 | 1 | 1 |
| Simochromis babaulti | 2 | 1 | 4 | 3 | 0.8751 | 1.00 | 3.70 | 1 | 1 |
| Spathodus erythrodon | 1 | 1 | 1 | 1 | 0.3979 | 3.00 | 4.50 | 1 | 0 |
| Spathodus marlieri | 1 | 1 | 4 | 3 | 0.4771 | 2.50 | 5.00 | 1 | 1 |
| Tropheus brichardi | 1 | 1 | 4 | 2 | 1.0969 | 2.50 | 4.70 | 1 | 1 |
| Tanganicodus irsacae | 1 | 1 | 1 | 1 | 0.3979 | 1.00 | 4.50 | 1 | 0 |
| Tropheus moorii | 1 | 1 | 2 | 1 | 0.3979 | 3.00 | 5.00 | 1 | 1 |
| Triglachromis otostigma | 1 | 1 | 1 | 1 | 1.4771 | 4.00 | 1.00 | 1 | 0 |
| Xenotilapia flavipinnis | 1 | 1 | 1 | 1 | 1.1903 | 4.00 | 3.30 | 1 | 0 |
| Xenotilapia ochrogenys | 2 | 1 | 2 | 2 | 1.0211 | 4.00 | 2.80 | 1 | 1 |
| Xenotilapia spilopterus | 1 | 1 | 1 | 1 | 1.1761 | 5.00 | 3.00 | 1 | 0 |
